# Impact of an Education Training Package to Anganwadi Workers for Improving Oral Health Knowledge Among Mother-Child Dyads in Kerala, India: Protocol for a Mixed Methods Implementation Study

**DOI:** 10.2196/91171

**Published:** 2026-05-08

**Authors:** Venkitachalam Ramanarayanan, Chandrashekar Janakiram, Vineetha Karuveettil, Sreelakshmi Mohandas, Das P Anaswara

**Affiliations:** 1 Public Health Dentistry Amrita School of Dentistry Amrita Vishwa Vidyapeetham Kochi, Kerala India; 2 Community Medicine Amrita School of Medicine Amrita Vishwa Vidyapeetham Kochi, Kerala India

**Keywords:** community health workers, oral health, implementation science, India, preschool child

## Abstract

**Background:**

Maternal and child oral health remains a significant public health concern in India, contributing to early childhood caries, adverse pregnancy outcomes, and long-term health complications. Anganwadi workers (AWWs), under the Integrated Child Development Services Scheme (ICDS), are well positioned to promote preventive oral health behaviors. However, oral health education is not part of their core training. This protocol outlines an implementation research study to develop, deliver, and evaluate a contextualized oral health education training package through AWWs for mother-child dyads in Kochi, Kerala.

**Objective:**

The objectives of this study are to (1) assess the current oral hygiene knowledge, attitude, and behavior and identify barriers and facilitators for implementing the oral health education training package among AWWs, parents, and other key stakeholders; (2) evaluate the oral health education training package for coverage, acceptability, adoption, fidelity, and scalability; and (3) assess the impact of an oral health education training package on the improvement in oral health knowledge, attitude, and behavior among mothers.

**Methods:**

A mixed methods implementation study will be conducted across 100 Anganwadi centers in 3 phases following the Exploration, Preparation, Implementation, and Sustainment framework. Phase 1 will assess baseline oral hygiene practices and identify contextual barriers and facilitators using structured questionnaires and qualitative interviews. In phase 2, AWWs will be trained using a structured oral health education package and will deliver oral health messages to mother-child dyads during routine sessions. Implementation outcomes—acceptability, coverage, adoption, fidelity, and scalability—will be evaluated using observation checklists and program tracking tools. Phase 3 will assess the impact by evaluating changes in maternal oral health knowledge, attitude, and behavior through preintervention and postintervention questionnaires administered to a subsample of mothers. Dissemination of findings and planning for long-term integration into ICDS will also be undertaken.

**Results:**

The study obtained funding in May 2024. The data collection commenced in April 2025. A total of 101 Anganwadi centers have been enrolled. Permissions, stakeholder meetings, baseline data collection, preparation of the teaching module, and training of AWWs (September 2025) have been completed. Follow-up visits to assess implementation parameters are ongoing. Data collection is expected to be completed in April 2026, followed by data analysis in May 2026 and dissemination by July 2026.

**Conclusions:**

This study is expected to generate practical insights into the feasibility of integrating oral health education into ICDS through AWWs. The intervention will be embedded within existing ICDS touchpoints and supported by centralized training, travel allowances, and regular supervision. Findings are expected to inform a scalable, community-based model aligned with national policy priorities for oral health promotion.

**Trial Registration:**

Clinical Trial Registry of India CTRI/2025/07/090759; https://tinyurl.com/32yu6avz

**International Registered Report Identifier (IRRID):**

DERR1-10.2196/91171

## Introduction

Oral health is essential for overall health, and poor oral hygiene can lead to various oral diseases, affecting both children and adults [1-3]. Maternal and child oral health is of particular concern as it plays a crucial role in early childhood development and sets the foundation for long-term oral health. Maternal oral health has been linked to adverse pregnancy outcomes, such as preterm birth, low birth weight, and increased risk of early childhood caries (ECC) in children [4]. ECC, a preventable disease, affects infants and toddlers, causing pain, infections, and long-term dental problems. Poor oral health in early childhood can impact a child’s growth, nutrition, speech development, and school performance [5]. Oral health and nutrition are closely linked in early childhood, as dietary habits affect both growth and dental development. Poor nutrition, particularly high sugar intake and inappropriate feeding, increases the risk of ECC and impacts overall health [6,7]. Thus, addressing maternal and child oral health is crucial for promoting well-being and reducing the burden of oral diseases.

Despite the burden, inequity exists in the availability and accessibility of professional oral health services. Oral care at the grassroots level is limited, necessitating the need for task shifting to less specialized health care workers by providing appropriate training and supervision [1]. Anganwadi workers (AWWs) are community-based health workers under the Integrated Child Development Services Scheme (ICDS) of the Government of India. They are primarily women selected from the local community and are trained to deliver maternal and child health care services at the grassroots level. In addition to educating children and mothers at the designated Anganwadi centers (translated as courtyard shelters), they also provide prenatal and postnatal education through home visits and conduct regular health checkups for pregnant women. They monitor the growth and development of children aged younger than 6 years. As of 2023, there were approximately 1.34 million AWWs in India [8]. They have a unique opportunity to reach mothers and children in rural and underserved areas, where access to dental care may be limited, and can play a key role in delivering oral health education interventions. Integrating oral health education with the regular nutrition and health sessions conducted by AWWs under ICDS provides a feasible, sustainable approach to reduce ECC and promote healthy growth [9,10]. Similar studies have shown that such interventions contributed to improved oral health knowledge, attitudes, and behaviors among mothers and children, as well as improved use of dental services [11-13].

Despite this evidence, oral health has historically received limited attention in national health agendas and policies [14]. Only in recent years has it begun to gain recognition as an integral component of overall health and well-being. The National Oral Health Program (NOHP) was piloted in 1999. However, it was in 2013 that the program was revamped to its present form [15]. One of the activities under NOHP includes oral health promotion through training of dental and paradental health functionaries in the health care delivery system. Anecdotal evidence suggests that the NOHP is yet to be fully implemented in Kerala, with the current focus being more on setting up dental health care units. It is not well established whether AWWs have received standardized or formal training for oral health promotion [16,17].

Few studies have explored the effectiveness of oral health promotion by AWWs in India [16-20], but the focus on implementation parameters has been limited. This study addresses a critical gap by evaluating a model for integrating oral health into community-based maternal and child health services through Anganwadi centers**.** This study is an implementation research project aimed at evaluating the feasibility and implementation of an oral health education training package delivered through AWWs using the Exploration, Preparation, Implementation, and Sustainment (EPIS) framework. Changes in oral health knowledge among mother-child dyads will be assessed as secondary exploratory effectiveness outcomes. The objectives of this implementation research are as follows:

- To assess the current oral hygiene knowledge, attitude, and behavior and identify barriers and facilitators for implementing the oral health education training package among AWWs, parents, and other key stakeholders
- To evaluate the oral health education training package for coverage, acceptability, adoption, fidelity, and scalability
- To assess the impact of an oral health education training package on the improvement of oral health knowledge, attitude, and behavior among mothers

## Methods

### Study Design

This implementation science project adopts a sequential mixed methods design and will be conducted in 3 distinct but interrelated phases. The overarching aim is to develop, implement, and evaluate a contextualized oral health education training package for AWWs in India.

**Figure 1:**
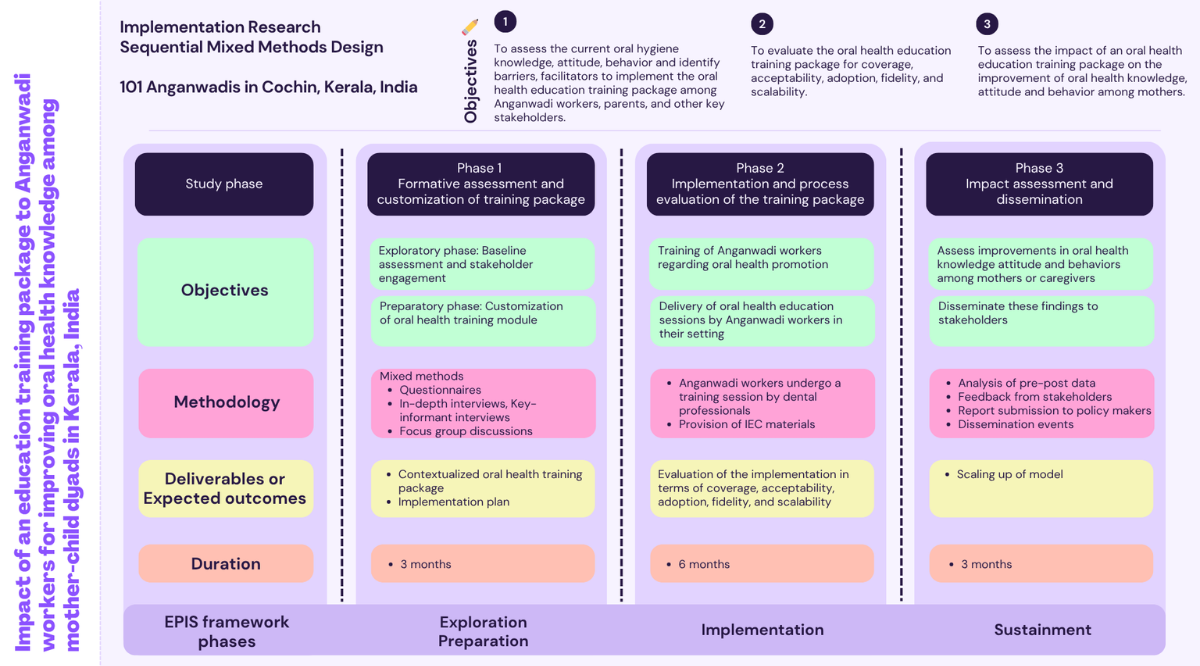
Study framework. EPIS: Exploration, Preparation, Implementation, and Sustainment; IEC: information, education, and communication.

This implementation research is guided by the EPIS framework—exploration, preparation, implementation, and sustainment [21]—which provides a structured approach for adopting evidence-based practices within real-world systems (Figure 1). The study will be undertaken in 3 phases: formative assessment and customization of the training package (phase 1), implementation and process evaluation of the training package (phase 2), and impact assessment and dissemination for scaling up (phase 3).

### Study Setting

The study will be implemented in Anganwadi centers in Kochi, Kerala, India.

### Participants and Eligibility

Participants will be recruited across 3 study phases and will include AWWs, ICDS supervisors, Child Development Project Officers (CDPOs), and mothers or caregivers of children enrolled in Anganwadi centers. The inclusion criteria for each group are presented in Textbox 1.

**Inclusion criteria for study participants.**

**Anganwadi workers**

- Anganwadi workers currently working at Anganwadi centers located within the Kochi Urban III administrative region and expected to continue serving in the study centers during the study period

**Mothers or caregivers**

- Mothers or primary caregivers whose preschool children (aged 1-6 years) are enrolled beneficiaries of any Anganwadi center in Kochi Urban III administrative region and who are able to attend Anganwadi sessions and provide informed consent

**Child Development Project Officers (CDPOs)**

- CDPOs currently working in Ernakulam District who oversee sectors included in the study

**Integrated Child Development Services Scheme (ICDS) supervisors**

- ICDS supervisors currently supervising the selected Anganwadi centers within the Kochi Urban III administrative region

### Implementation Framework

The EPIS framework will be incorporated during the various phases of the study. Phase 1 aligns with exploration and preparation, phase 2 corresponds to implementation, and phase 3 aligns with sustainment.

#### Phase 1: Formative Assessment and Customization of the Training Package (Exploration and Preparation)

Dental diseases in childhood, especially ECC, are a major public health concern globally [22]. In India, nearly half of all children are affected, and in Kerala, ECC prevalence among preschoolers ranges from 44% to 53% [22,23]. This underscores the urgent need for preventive interventions at the early childhood stage. However, information is limited on both AWWs’ knowledge of oral health and that of the children’s parents and caregivers.

The objective of this phase is to assess existing oral hygiene knowledge, attitude, and behavior and implementation readiness through surveys, interviews, and focus group discussions (FGDs) with mothers, AWWs, and ICDS officials (CDPOs and ICDS supervisors).

Findings obtained from the exploratory stage will guide the preparatory stage, which involves cultural and linguistic adaptation of the NOHP oral health manual. This will include developing and customizing training and information, education, and communication (IEC) materials, as well as formulating a context-specific implementation plan for this oral health education training package. The manual covers key aspects of oral health, including common oral diseases and their prevention in home and professional settings, management strategies for oral diseases, handling dental emergencies, debunking common myths, sharing facts about dental health, and interactive activities to reinforce learning and understanding of concepts.

#### Data Collection in Phase 1

Quantitative and qualitative methods will be used to achieve these objectives, including questionnaires, in-depth interviews, key informant interviews, and FGDs with stakeholders such as AWWs, government officials, and mothers, parents, and caregivers of children. In the quantitative phase, a pretested questionnaire developed from previous literature [24-27] will be used to assess current oral hygiene knowledge, attitude, and behavior among purposively selected parents and caregivers, and the results will be analyzed descriptively.

In the qualitative phase, purposive sampling will be used. Separate in-depth interview guides and FGD guides will be developed for each participant group, including AWWs, mothers, ICDS supervisors, and CDPOs (administrative officers). FGDs will be conducted with AWWs and mothers until theoretical saturation is reached. In-depth interviews will be carried out with mothers and AWWs, while key informant interviews will be conducted with CDPOs and ICDS supervisors of the selected Anganwadi centers. All qualitative data will be audio-recorded, transcribed, translated, and thematically analyzed using a deductive approach.

Findings from both phases will be used to customize the training manual on oral health promotion for health workers developed by the Centre for Dental Education and Research, All India Institute of Medical Sciences (AIIMS), for the NOHP, Ministry of Health and Family Welfare, Government of India [28] and to formulate an implementation plan to guide the structured delivery of the oral health training and education package. A contextualized training module for AWWs and a structured implementation plan detailing strategies for training delivery, monitoring, and integration into existing ICDS activities are expected to be achieved at the end of this phase.

#### Phase 2: Implementation and Process Evaluation of the Training Package (Implementation)

The focus is on training AWWs at the center level. Each Anganwadi center is staffed by one AWW and an assistant or helper; hence, implementation will be evaluated at the center level, with the AWW as the primary delivery agent.

The training package developed in phase 1 will be delivered by dental professionals or health personnel from the local health department. Each AWW will participate in a structured interactive session using PowerPoint presentations, demonstrations, and other teaching aids. IEC materials—including an oral health manual, display posters, and tooth models—will be provided to every Anganwadi center. Following the training, AWWs will be encouraged to integrate oral health education into their regular interactions with children, mothers, and expectant mothers. Flexibility will be allowed in how sessions are conducted so that AWWs can adapt delivery to their existing schedules and community context.

Evaluation of the oral health education training package will be done for coverage, acceptability, adoption, fidelity, and scalability over 6 months (Table 1).

**Table 1:** Monitoring and evaluation framework.

Outcome	Definition or measure	Assessment	Indicator
Acceptability	Attendance: proportion of AWWs^a^ attending the training program Willingness: proportion of Anganwadi centers initially agreeing to participate	Attendance sheet Consent form	Attendance: good (>75%), fair (25%-75%), and poor (<25%) Willingness: good (>75%), fair (25%-75%), and poor (<25%)
Coverage	Proportion of Anganwadi centers implementing the training package by conducting at least one session of a minimum of 30 min, validated through field assistants’ visits to the centers on the scheduled day of training or by reviewing video recordings of the sessions submitted by the AWWs	Field assistants’ direct visits to the Anganwadi centers on the scheduled day of training Ongoing contact with the AWWs Review of video recordings of the sessions submitted by the AWWs	Good: >70% of centers Average: 20%-70% of centers Poor: <20% of centers
Adoption	Proportion of Anganwadi centers conducting more than one training session within 6 mo	Documents (registers and/or videos)	Good: >50% of centers conduct multiple sessions Average: 25%-50% of centers conduct multiple sessions Poor: <25% of centers conduct multiple sessions
Adoption	Total number and duration of sessions conducted by each center	Number, hours	—^b^
Fidelity	Proportion of Anganwadi centers adhering to the training module as originally outlined in the manual and training sessions	Direct observation by field assistants	Good: >50% of centers Poor: <50% of centers

^a^AWW: Anganwadi worker.

^b^Not applicable.

#### Phase 3: Impact Assessment and Dissemination for Scaling Up

This phase focuses on evaluating and sharing the outcomes of the implemented training package. The objective is to assess improvements in oral health knowledge, attitude, and behaviors among mothers and caregivers, and to disseminate these findings to stakeholders. Analysis of preintervention and postintervention oral health knowledge, attitude, and behavior scores will be conducted using the questionnaire used in phase 1 from a subsample of beneficiaries from each Anganwadi center.

The study findings will be disseminated to multiple stakeholders through summary briefs and oral presentations to state-level and district-level ICDS officials—such as the ICDS District Officer, CDPOs, and ICDS supervisors—to support integration of the oral health education training package into routine ICDS activities. Outputs will also be shared with AWWs through local feedback sessions at the sector level, providing practical recommendations for sustaining oral health education within their existing schedules.

### Sample Size

A total of 101 Anganwadi centers in the Kochi Urban III administrative region that include the field practice area of the implementing institution, will be selected. Each Anganwadi unit typically covers about 1000 people, of whom about 15 to 30 are children. The intended beneficiaries of the project would be approximately 1500 to 3000 children and their mothers directly, and about 6000 family members indirectly. The Anganwadi centers hold regular contact sessions (at least twice monthly) with the mothers and families of these children. Thus, the project envisions improving awareness and practices in oral health as a cascading effect from AWWs to children and mothers and ultimately to their families and community.

For the assessment of change in oral health knowledge among mothers, a subsample of approximately 10% of mothers of enrolled children will be selected for the pre-post assessment. This sample size is expected to be sufficient to detect a moderate improvement in knowledge scores (effect size approximately 0.4-0.5) with 80% power at a 5% significance level in paired measurements. As participants are clustered within Anganwadi centers, the analysis will account for clustering using cluster-adjusted statistical methods (eg, mixed effects models or cluster-robust SEs).

### Data Analysis

Data analysis will be undertaken in line with the sequential mixed methods design of the study, with quantitative and qualitative findings analyzed separately and subsequently integrated to inform intervention adaptation and refinement of implementation strategies.

#### Phase 1

Descriptive statistics will be used to summarize participant baseline characteristics and outcome measures. Continuous variables, such as oral health knowledge, attitude, and behavior scores, will be reported as means and SDs or medians and IQRs, as appropriate. Qualitative data will be transcribed verbatim, translated where required, and analyzed using a thematic analysis approach. Coding will be conducted iteratively, with themes developed across stakeholder groups. Data will be managed using QDA Miner Lite (Provalis Research).

#### Phase 2

Implementation outcomes, including coverage, acceptability, adoption, fidelity, and scalability, will be calculated based on criteria described in Table 1 and expressed as frequency and percentages.

#### Phase 3

To evaluate changes between preintervention and postintervention assessments, linear mixed effects regression models will be used. Time (preintervention assessment vs postintervention assessment) will be included as a fixed effect. Random intercepts will be specified for both Anganwadi centers and individual participants to account for clustering of participants within centers and within-participant correlation arising from repeated measurements. Maternal age, education level, and occupation variables will be included as fixed effects where appropriate to adjust for possible confounding. Knowledge, attitude, and behavior scores will be analyzed as separate outcome variables. Missing data will be assessed for extent and pattern. Mixed effects models allow the inclusion of participants with incomplete outcome data under the missing at random assumption. If substantial missing data are observed, sensitivity analyses using multiple imputation will be considered. Effect estimates will be reported with 95% CIs, and statistical significance will be set at *P*<.05. Analysis will be done using SPSS software (version 22; IBM Corp).

### Dissemination

In addition to the dissemination of the study results to the stakeholders as outlined in phase 3, scientific dissemination through peer‑reviewed journals and presentations at national and international conferences is planned. This is intended to support future scale-up by providing evidence‑based documentation of the model’s feasibility and implementation outcomes, identifying barriers and facilitators to embedding oral health promotion within ICDS, and offering a ready-to-adapt training and monitoring framework that can be replicated in other districts or states in India. If the implementation outcomes are favorable, the model will be proposed for incorporation into the routine functioning of ICDS.

### Ethical Considerations

The study has been approved by the institutional ethics committee of Amrita Institute of Medical Sciences, Kochi (ECASM-AIMS-2024-454). Permission has been obtained from the ICDS District Officer, Government of Kerala, and approval has been received from the Health Ministry Screening Committee, Government of India, and is registered with the Clinical Trial Registry of India (CTRI/2025/07/090759).

Written consent to participate will be obtained from all participants (AWWs, ICDS supervisors, CDPOs, and mothers). Participants will receive a plain language information sheet outlining the details of the study. Contact details will be collected solely for administrative purposes (eg, scheduling and follow-up). No personal identifiers will be included in study records, analysis files, or publications, and all data will be anonymized. All data will be stored on a secure, encrypted drive with restricted access to the principal investigator and authorized research staff.

An honorarium to the participating Anganwadi teachers will be provided for undertaking oral health education modules. Travel allowances will also be provided for Anganwadi teachers to attend training session and other meetings.

## Results

The grant application was approved in May 2024, following which the regulatory approvals were obtained. A total of 101 AWWs, representing their respective Anganwadi centers, were enrolled in the study. Needs assessment meetings were held in April 2025. Baseline data collection (July-August 2025) has been completed among selected AWWs, mothers, and ICDS officials using questionnaires, FGDs, and in-depth interviews.

Findings from the formative qualitative phase informed the contextual adaptation and development of the oral health training manual for AWWs. Subsequently, 91 (90.1%) AWWs from Anganwadi centers in Ernakulam district completed a centralized 3-day training program conducted by dental experts (September 2025). IEC materials and oral health manuals were distributed to all trained AWWs.

As of February 2026, the trained AWWs are actively delivering oral health education to mothers and preschool children at their respective centers. Ongoing monitoring and evaluation of implementation outcomes, including coverage, adoption, and fidelity, are underway. The study team anticipates completion of all study phases by April 2026, followed by analysis in May 2026 and completion of a detailed report and manuscript writing by July 2026. Dissemination through conference presentations and stakeholder policy briefings is also expected between June and July 2026.

## Discussion

### Expected Outcome

The proposed study is expected to provide important evidence on the implementation and effectiveness of an oral health education training package delivered through AWWs. The ICDS program, launched in 1975 by the Government of India, was envisioned as a comprehensive initiative to address the health, nutrition, and early education needs of children aged younger than 6 years, along with pregnant and lactating women. AWWs are the cornerstone of this initiative, serving as community-based agents of change.

However, as far as the introduction of a new component (oral health promotion) is concerned, several practical and operational considerations must be acknowledged in the planning and execution of this intervention to ensure its success and scalability. AWW service delivery is influenced by a complex interplay of means, opportunity, and motives [29], which can help contextualize many of the practical barriers anticipated in this study.

### Training Content and Knowledge Gaps

Oral health education is not part of the core ICDS training, and AWWs differ in their baseline knowledge, public communication skills, and confidence levels. Therefore, variability in how they deliver oral health messages to mothers is anticipated. These differences emphasize the need for a user-friendly, linguistically appropriate, and visually rich training manual. The project uses the official training manual of the NOHP, developed by the Ministry of Health and Family Welfare, incorporating simplified and regionally contextualized content. This approach is supported by evidence from a study conducted in Bihar [30] that found that well-trained frontline workers were more likely to deliver essential services effectively.

### Workload and Coordination Challenges

AWWs perform multiple duties ranging from growth monitoring and preschool education to home visits and nutrition distribution, which can limit their bandwidth to take on new tasks [29,30]. Findings from an evaluation of ICDS in Gujarat [31] reported that staff workload, along with inadequate training and supervision, affected service delivery. To avoid further overburdening AWWs, training for oral health will be scheduled during existing sector or project meetings, with support from ICDS officials. Travel allowances and centralized training venues are also planned to enhance participation.

### Variability in Motivation and Fidelity

Differences in motivation, influenced by recognition, incentives, and perceived relevance of the new task, may impact the fidelity of intervention delivery. Evidence shows that frontline workers perform better when supported by feedback, supervision, and tangible acknowledgment of their efforts [30]. This project will address these aspects by ensuring regular monitoring, feedback, and recognition during ICDS review sessions or through a separate review session.

### Constraints With Participation of Mothers

Mothers, the primary beneficiaries of the program, may have difficulty attending sessions due to domestic responsibilities, work obligations, or low prioritization of oral health. In a community-based controlled trial involving Anganwadi centers, it was shown that embedding health education into existing sessions improved reach and impact [19]. Therefore, this study will integrate oral health education into existing touchpoints such as immunization days and monthly mother-AWWs meetings to optimize attendance.

### Monitoring, Behavior Change, and Data Validity

Evaluating changes in knowledge and behavior poses methodological challenges, particularly when relying on self-reported measures from low-literacy populations. A mixed methods design using preintervention and postintervention surveys, interviews, and FGDs allows both quantifiable and contextual data to be captured. This triangulated approach will better document the intervention’s impact, especially regarding improved maternal awareness and oral health behaviors such as brushing frequency and dietary choices.

### Scalability, Policy Alignment, and Relevance to the ECC Burden

Although this is a pilot study covering 101 Anganwadi centers, the findings aim to inform broader, scalable models for oral health integration into ICDS. The study supports the NOHP’s policy direction, which encourages oral health promotion through nondental health workers. The training manual developed by the Centre for Dental Education and Research, AIIMS, in 2018 explicitly recognizes AWWs and schoolteachers as vital links for grassroots oral health education [28]. Yet, a nationwide implementation gap persists, especially in Kerala, where NOHP efforts have focused more on setting up dental units rather than health promotion [32]. This study will address that gap by integrating a preventive oral health component into an existing, trusted community-based system.

The prevalence of ECC remains high in India [23], particularly in underserved and low-income populations. ECC can result in pain, infection, poor nutrition, and school absences and contributes significantly to the early burden of disease. Interventions like this, delivered through trusted community workers and targeting mothers and young children, may offer a practical and sustainable approach to reducing ECC rates through improved awareness and preventive behaviors [19].

## Appendix

Multimedia Appendix 1 Peer-review reports from the Borrow Foundation (Hampshire, UK).

## Abbreviations

AIIMS: All India Institute of Medical Sciences
AWW: Anganwadi worker
CDPO: Child Development Project Officer
ECC: early childhood caries
EPIS: Exploration, Preparation, Implementation, and Sustainment
FGD: focus group discussion
ICDS: Integrated Child Development Services Scheme
IEC: information, education, and communication
NOHP: National Oral Health Program
