# Supplementary material for: Impact of an Education Training Package to Anganwadi Workers for Improving Oral Health Knowledge Among Mother-Child Dyads in Kerala, India: Protocol for a Mixed Methods Implementation Study
Source: JMIR Res Protoc. 2026 May 8;15:e91171. doi: 10.2196/91171 (PMC13197742; doi:10.2196/91171)
Supplement: Multimedia Appendix 1 [file resprot_v15i1e91171_app1.pdf]

The Borrow Foundation

Padnell Grange, Padnell Road, Cowplain, Waterlooville, Hampshire, P08 8ED, United Kingdom
Tel: +44 (0)23 9226 2222 Fax: +44 (0)23 9224 1401 enquiries@borrowfoundation.org www.borrowfoundation.org

Proposal Review

Transforming Oral Health: Empowering Community-based Health (Anganwadi) Workers to Improve Awareness and Practices in Mothers, Children, and Families

This is an interesting proposal and something on which the National Governments in many countries are actively working also broadly. I have tried to provide a detailed review by answering the questions below:

- **What is the potential importance of the project in terms of its scientific merits, originality and potential impact?**

  The project holds average importance in terms of scientific merit as there is no specific research activity which will be undertaken. I cannot say that this is a novel concept as government of India is developing and delivering training modules for frontline health workers. There are plenty of training modules that exist locally and I am not sure why the investigators want to develop another training programme. The problem is that how these trainings are implemented and integrated is a not known and I think this is where the investigators should have focussed on rather than doing needs assessment. The impact can be substantial if the focus is on implementation rather than training module development.

- **Are the research questions, aims and hypothesis clearly expressed and realistic?**

  The aims and objectives are adequately explained and written well. Whether these are realistic and can be achieved in one year is difficult to predict.

- **Are the study design and methods appropriate?**

  As I had mentioned above, I do not see a reason to again undertake a needs assessment or the need to develop another training module. The frontline health workers already have got existing modules on oral health developed by National Health Mission of India. The focus should have been to adopt this to the local setting, implement it and check its applicability.

- **Is the statistical / analytical design appropriate and clearly explained?**

  The intent of analysis is clearly explained but what kind of analysis will be undertaken is not mentioned in the proposal. There are too many objectives for a one-year study. Also, the expanse or the sampling frame of urban health centers and the population they cater to is explained but no further explanation of sample size calculations for needs assessment is provided.

- **Are the primary and secondary outcomes clearly defined?**

  Not really. I am not very sure what will be the final outcome be or what are the indicators of success/ failure the investigators will be using.

- **Do the researchers appear to have the appropriate experience, qualifications, and competence to undertake the proposed study / project?**

  The research team is very competent and well equipped to undertake the project.

- **Are the funding requirements and proposed timeframe realistic and justifiable?**
  The funding requirement is appropriate but the time frame in which the researchers are trying to achieve these objectives is very ambitious.

- **In conclusion, what are the strengths and weaknesses of the application. Does the study / project appear to be viable /feasible and are the findings likely to add a new element to existing knowledge or practice?**

  Strengths of the application: very good and competent team, access to an urban health centre for project roll out, understanding of health systems by the team.

  Weaknesses: too ambitious, training module already exists, less focus on implementation, analytical design not explained well.

  Given the experience of the team and access to a study area, the project appears to be feasible however, I am not too sure how the findings will add new elements to the practice. I think if the focus would have been on implementing the existing resources and checking their acceptability or barriers in uptake, it would have made a very significant impact.
